# Role of respondents’ education as a mediator and moderator in the association between childhood socio-economic status and later health and wellbeing

**DOI:** 10.1186/1471-2458-14-1172

**Published:** 2014-11-18

**Authors:** Mashhood Ahmed Sheikh, Birgit Abelsen, Jan Abel Olsen

**Affiliations:** Department of Community Medicine, University of Tromsø, Tromsø, NO 9037 Norway

**Keywords:** Norway, Childhood socioeconomic conditions, EQ-5D, Self-rated health, Wellbeing, Mediation, Effect decomposition

## Abstract

**Background:**

Most research assessing the effect of childhood socioeconomic status (CSES) on health in adulthood has focused on cause-specific mortality. Low CSES is associated with mortality from coronary heart disease, lung cancer, and respiratory diseases in adulthood. But little evidence is available on the unique effect of different indicators of CSES on subjective measures of health and wellbeing in adulthood.

**Methods:**

Cross-sectional data from the last wave of The Tromsø Study (n = 12,984) was used to assess the unique effect of three indicators of CSES (childhood financial conditions, mothers’ education and fathers’ education) on a range of subjective health measures: EQ-5D health dimensions, self-rated health, age-comparative self-rated health, as well as subjective wellbeing. Data was analyzed with the Paramed command in Stata. Log-linear regression was used for the subjective measures of health and wellbeing to estimate the natural direct effects (NDE’s), natural indirect effects (NIE’s), controlled direct effects (CDE’s) and marginal total effects (MTE’s) as risk ratios (RRs).

**Results:**

Low childhood financial conditions were associated with lower health and wellbeing in adulthood, independently of respondents’ education. Among men, Low childhood financial conditions increased the risk (NDE) of being unhealthy on the composite EQ-5D by 22% (RR 1.22, 95% 1.14-1.31) and on subjective wellbeing by 24% (RR 1.24, 95% 1.18-1.30), while for women the risk increased by 16% (RR 1.16, 95% 1.10-1.23) and 26% (RR 1.26, 95% 1.19-1.33), respectively. Among men, the NDE of low mothers’ education on age-comparative self-rated health increased by 9% (RR 1.09, 95% 1.01-1.16), while the NIE increased the risk by 3% (RR 1.03, 95% 1.01-1.04). The NDE of low mothers’ education increased the risk on anxiety/depression among women by 38% (RR 1.38, 95% 1.13-1.69), whereas the NIE increased the risk by 5% (RR 1.05, 95% 1.02-1.08).

**Conclusions:**

Childhood financial conditions have a unique direct effect on a wide range of health and wellbeing measures. These findings apply to both men and women. Generally, parental education has an indirect effect on later health, but mothers’ education may also have a long-term direct effect on later health.

## Background

The commonly used indicators of childhood socio-economic status (CSES) can be categorized into two groups: indicators of social background (e.g. mothers’/fathers’ education), and indicators of economic background (e.g. mothers’/fathers’ income, home ownership, housing characteristics, etc.) [1–3]. Galobardes et al. [4, 5] reviewed 40 studies assessing the association between CSES and mortality, and showed that low CSES was associated with mortality from coronary heart disease, lung cancer, stomach cancer and respiratory diseases in adulthood [4]. Several studies exploring the association between CSES and health in adulthood [4–6] have analyzed whether SES in adulthood (ASES) has a mediating role, i.e. CSES effects ASES, which in turn has an effect on health in adulthood (conceptualized as the indirect effect), or whether the CSES has an independent effect on health in adulthood, i.e. not mediated by ASES (conceptualized as the direct effect). One review showed a general effect of CSES on health in adulthood, but the estimates were attenuated after adjusting for ASES, indicating that a direct effect does exist between CSES and later health, but that some of this effect may be mediated by ASES [4].

There are caveats. Most studies included in the aforementioned reviews used indicators of economic background to assess CSES, and therefore very little evidence is available about the effect of the indicators of social background on health and wellbeing in adulthood [7–11]. High CSES may provide the opportunity to flourish later in life, not only through higher education and income, but also better health. A higher social background in terms of high parental education is likely to inspire children to pursue higher education. However, it is uncertain whether social background alone (i.e. independent of the economic conditions) has a long-term effect on later health and wellbeing. Previous research has indicated that the causal mechanisms of economic and social background on health later in life are likely to be different [3, 7]. In the Helsinki Health Study, Mäkinen et al. [7] studied the effect of mothers’/fathers’ education and self-reported economic difficulties experienced before 16 years of age on self-reported adult physical and mental functioning. They found no direct effect of mothers’ and fathers’ education on adult physical or mental functioning, but they found a direct effect of economic difficulties in childhood on both adult mental and physical functioning [7]. Other studies have indicated that different indicators of social background in childhood have different effects on later health [2, 8]. Mothers’ education is more important than fathers’ education for health in adulthood, and this effect is mediated by the respondent’s education, i.e. high mothers’ and fathers’ education is associated with high respondents’ education, which in turn is associated with better health [2, 8]. This is in contrast to most previous studies [4, 5, 12], in which evidence of a direct effect of CSES on health in adulthood was found using indicators of economic background to assess CSES.

Most previous studies included only one indicator of CSES [13], so the unique effects of social and economic indicators of CSES on health in adulthood could not be analyzed or compared [14]. Since indicators of CSES may be correlated, it is not clear whether different social and economic indicators of CSES have an independent effect on health in adulthood [1, 14].

While many studies have analyzed the effect of CSES on cause-specific mortality and cardiovascular disease [4, 5, 15, 16], little evidence is available about the effect of CSES on subjective measures of health and wellbeing in adulthood, like self-rated health [6, 11, 17–20], wellbeing [21], and psychosocial functioning [16, 17]. Some studies have assessed the predictive effect of CSES on functional limitation [20, 22], allostatic load [23] and psychosocial functioning [2, 10, 16, 17, 21, 22, 24–28], but the results were not consistent. Moreover, previous studies have shown that self-rated health is an unreliable measure of health [29, 30]. Therefore, it is important to analyze and report different measures of health to assess the sensitivity of the estimates.

The aim of this paper is to estimate and compare the direct and indirect influence (mediated by respondents’ education) of three indicators of CSES: childhood financial conditions, mothers’ education, and fathers’ education, on: i) the health dimensions included in the EQ-5D; ii) self-rated health; iii) age-comparative self-rated health, and; iv) subjective wellbeing.

## Methods

### Study population

The Tromsø Study is a prospective cohort study of the population residing in the municipality of Tromsø. With more than 70,000 inhabitants, Tromsø is the largest city in Northern Norway. It is situated at 69°N, approximately 400 km north of the Arctic Circle. Between 1974 and 2007/2008, six waves of the Tromsø Study were conducted (referred to as Tromsø I-VI). The current paper is based on data from the latest wave: 19,762 subjects were invited and sent a study questionnaire by post; 12,984 (65.7%) returned the questionnaire (6,054 men and 6,930 women, born between 1920 and 1977). The sample of 19,762 was selected by inviting total birth cohorts born in 1920–1947 (aged 60–87), 40% of the total birth cohorts born in 1948–1954 (aged 53–59), 1955–1959 (aged 48–52), and 1960–1964 (aged 43–47), total birth cohorts born in 1965–1967 (aged 40–42) and 10% of the total birth cohort born in 1968–1977 (aged 30–39) [31]. The study design and characteristics of the study sample have been described previously [31].

### Measures of subjective health and wellbeing

Subjective health was assessed in the study questionnaire by the EQ-5D, self-rated health, and age-comparative self-rated health. The EQ-5D measures five health dimensions: mobility, self-care, usual activities, pain/discomfort, and anxiety/depression [32]. Each health dimension was separated into three levels: level one was described as ‘no problems’, level two as ‘some problems’ and level three as being ‘unable’ or having ‘extreme problems’. A composite EQ-5D binary variable was constructed by classifying respondents ticking level one for all five health dimensions as healthy, and the remaining as unhealthy. Respondents with missing values for any of the five health dimensions were excluded. Separate binary variables were constructed for each of the five health dimensions in the same manner as for the composite variable, i.e. by comparing respondents with ‘no problems’ to those with ‘some’ or ‘extreme’ problems.

Self-rated health was measured by the question “How do you in general consider your own health to be?” Possible responses were: very good, good, neither good nor bad, bad, and very bad. Those ticking very good or good were classified as healthy, and the remaining as unhealthy. Age-comparative self-rated health was measured with the question “How do you consider your health compared to that of others your age?” Possible responses were: much better, somewhat better, about the same, a little worse, and much worse. Those ticking the first two levels were classified as relatively healthy, and the remaining as relatively unhealthy.

Subjective wellbeing was measured by the first three items from the satisfaction with life scale [33]. These were “In most ways my life is close to my ideal”, “The conditions of my life are excellent”, and “I am satisfied with my life”, each measured on a 7-point scale from completely disagree (1) to completely agree (7). Those who reported 6 or 7 for all three items were considered to have high subjective wellbeing, and the remaining as having low subjective wellbeing.

### Indicators of CSES

Recall of CSES is expected to be fairly accurate [34]. The present analysis used self-rated childhood financial condition as the indicator of economic background, and was measured by the question, “How was your family’s financial situation when you were a child?” on a 4-point scale. Those who answered very good or good were considered to have a high childhood financial conditions, while those who answered difficult or very difficult were considered to have low childhood financial conditions.

Mothers’/fathers’ education was used as an indicator of social background, and were measured separately on a 5-level scale: primary and secondary school or similar (i.e. 7–10 years of schooling), vocational school, high school, college or university (less than 4 years), and college or university (4 years or more). If the first level was ticked, the respondent was classified as having low mothers’/fathers’ education, and the remaining as having high mothers’/fathers’ education.

### Indicator of ASES

Education of the respondents and their spouses, were measured by the same 5-level scale used for mothers’/fathers’ education. Those who replied positively to the first three levels (i.e. no college or university) were classified as having low education and the remaining as having high education. This classification differs from that of mothers’/fathers’ education due to a sharp increase in the duration of education across generations in Norway (Table 1).

**Table 1:** **Characteristics of the study sample (n = 12,984)**

Characteristics	N (%)	
**Sex** ^**a**^		
Male	6053	(46.6)
Female	6928	(53.4)
***Exposures***		
**Childhood financial conditions** ^**a**^		
Very good	699	(5.8)
Good	8011	(66.6)
Difficult	3113	(25.9)
Very difficult	204	(1.7)
**Mothers’ education** ^**a**^		
Primary and secondary school or similar 7–10 years	9233	(78.7)
Vocational school	1473	(12.6)
High school	338	(2.9)
College or University (less than 4 years)	500	(4.3)
College or University (4 years or more)	185	(1.6)
**Fathers’ education** ^**a**^		
Primary and secondary school or similar 7–10 years	7435	(64.2)
Vocational school	2480	(21.4)
High school	427	(3.7)
College or University (less than 4 years)	731	(6.3)
College or University (4 years or more)	507	(4.4)
***Mediator***		
**Respondents’ education** ^**a**^		
Primary and secondary school or similar 7–10 years	3673	(28.7)
Vocational school	3339	(26.1)
High school	950	(7.4)
College or University (less than 4 years)	2246	(17.5)
College or University (4 years or more)	2590	(20.2)
***Covariates***		
**Spouse’s education** ^**a**^		
Primary and secondary school or similar 7–10 years	2319	(23.9)
Vocational school	2815	(29.0)
High school	1061	(10.9)
College or University (less than 4 years)	1637	(16.9)
College or University (4 years or more)	1869	(19.3)
**Age (years)** ^**a**^		
30-39	509	(3.9)
40-49	3574	(27.5)
50-59	2436	(18.8)
60-69	4102	(31.6)
70-79	1829	(14.1)
80-89	531	(4.1)

^a^The total number does not add up to 12,984 due to missing values.

### Statistical analysis

Descriptive statistics were used to analyze the distribution of respondents by socio-demographic characteristics. Similarly, the distribution of respondents according to measures of subjective health and wellbeing and CSES was analyzed by cross tabulation and descriptive statistics. Stata ver. 13 was used for all statistical analysis.

**Figure 1:**
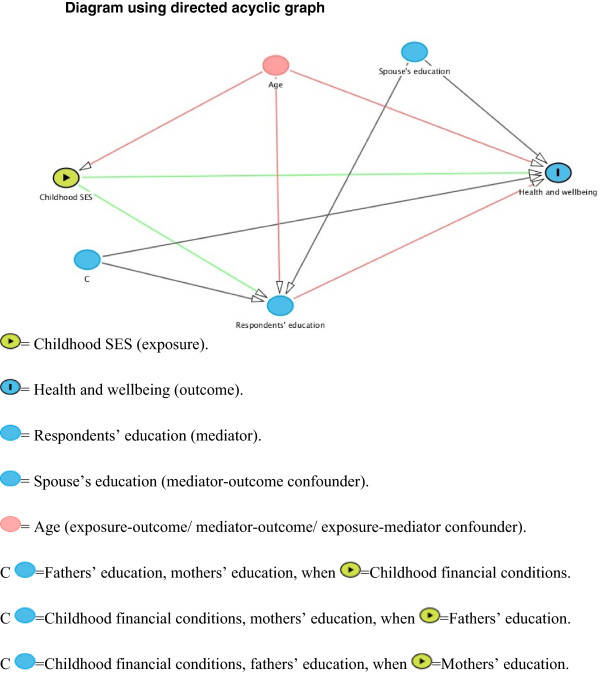
**Diagram using directed acyclic graph.**

Our aim was to estimate the natural direct effects (NDEs), controlled direct effects (CDEs) and natural indirect effects (NIEs) of self-rated childhood financial conditions, mothers’ education and fathers’ education on measures of subjective health and wellbeing after controlling for potential confounders. The assumed association between the variables is presented using a Directed Acyclic Graph [35, 36] (Figure 1). The direction of the arrows represents the direction of the effect. We hypothesized that the three indicators of CSES under investigation have a direct, as well as an indirect effect on health and wellbeing in adulthood.

Using the approach by Baron and Kenny [37], we assessed the associations between CSES and respondents’ education; respondents’ education and health and wellbeing; and CSES and health and wellbeing with linear regression and logistic regression models. The association between the indicators of CSES and subjective measures of health and wellbeing were statistically significant (p < 0.05), except the association between fathers’ education and subjective wellbeing. To assess the role of respondents’ education as a moderator, we further tested the interaction between each CSES indicator and respondents’ education, to see if the effect of CSES indicators on health and wellbeing in adulthood was homogenous across different levels of respondents’ education. We observed a statistically significant (p < 0.05) interaction between childhood financial conditions and respondents’ education, regressed on EQ-5D and subjective wellbeing. However, we did not observe a statistically significant interaction between childhood financial conditions and respondents’ education, regressed on self-rated health and age-comparative self-rated health.

The Paramed command in Stata [38] was used to perform mediation analyses, as it allows for exposure-mediator interaction. Furthermore, it can estimate NDEs, and NIEs in the presence of exposure-mediator interaction [39]. Logistic regression was used to analyze the effect of indicators of CSES on respondents’ education. Since the outcomes (unhealthy/low subjective wellbeing) were not rare, Log-linear regression was used to estimate the NDEs, CDEs, NIEs and marginal total affects (MTEs) as risk ratios (RRs) [39]. Each indicator of CSES constituted a separate exposure, to estimate the unique direct and indirect effect on health and wellbeing in adulthood, with 95% confidence intervals (CI).

As the exposures, mediator, and measures of subjective health and wellbeing were binary, the following models [39] fit the data, where y = health or wellbeing, a = CSES, m = respondents’ education, and c = covariates:
12

Separate analyses were conducted for each measure of subjective health and wellbeing, therefore respondents with missing values on CSES, respondents’ education, measures of subjective health and wellbeing, and covariates were excluded. In line with Valeri & VanderWeele [39], NDEs, the CDEs, and NIEs were estimated as RRs from model 1 and 2 as,


The two exposure levels being compared were a* =0 and a =1, where 0 = high CSES, and 1 = low CSES. The CDE expresses the effect of having low CSES on the outcome if the respondents’ education was controlled at a fixed level (either low or high education level), uniformly in the population. The NDE expresses how much the outcome (unhealthy/low wellbeing) would change if the exposure level were set at a =1 (low CSES) versus a* =0 (high CSES), but for each respondent, the mediator (respondents’ education) was kept at the level it would have had in the absence of the exposure (low CSES). The NIE expresses how much the outcome (unhealthy/low wellbeing) would change on average if the CSES were controlled at level a =1 (low CSES), but the mediator were changed from the level it would take if a* =0 (high CSES) to the level it would take if a =1. The MTE expresses how much the outcome would change overall for a change in exposure level from a* =0 to a =1. See Valeri & Vanderweele [39] for a detailed description of mediation analysis.

To estimate the CDEs, NDEs, NIEs and MTEs of financial conditions in childhood on measures of subjective health and wellbeing, those with high financial conditions in childhood were used as the reference group, and unhealthy/low subjective wellbeing was used as an outcome for all measures of health and wellbeing separately. To estimate the CDEs, NDEs, NIEs and MTEs of fathers’ education and mothers’ education, respectively, on health and wellbeing, those with high fathers’ and mothers’ education (high CSES), respectively, were used as the reference group, and unhealthy/low subjective wellbeing was used as an outcome for all measures of subjective health and wellbeing separately. Both fathers’ and mothers’ education were used as separate exposure variables. Statistically significant interaction (p < 0.05) was observed between the indicators of CSES and gender, regressed on the measures of subjective health and wellbeing, therefore the estimates are presented separately for men and women.

Previous studies have shown that parental education may only have an indirect effect on health in adulthood mediated by ASES [8–11]. Since some of the effect of parental education may be mediated by childhood financial conditions [40], we assessed whether this indicator was a mediator between mothers’/fathers’ education and respondents’ education, but the NIEs were RR ≈ 1.00 (null effect). Similarly, we assessed whether childhood financial conditions was a mediator between mothers’/fathers’ education and health and wellbeing in adulthood, and the NIEs (RR) were close to 1.00. Therefore, we ruled out the possibility that childhood financial conditions is a mediator-outcome confounder affected by mothers’/fathers’ education. Spouse’s education was associated with the three indicators of CSES and respondents’ education (data not shown). However, in order to bias the estimates, spouse’s education would have to be a mediator between CSES and health and wellbeing, so we assessed whether spouse’s education was indeed a mediator between these variables. Resultant the NIEs were RR ≈ 1.00; therefore, we ruled out the possibility that spouse’s education was a mediator-outcome confounder affected by CSES.

### Confounders

The identification of confounders was based on a priori knowledge of the association between the variables under study [41]. The diagram is illustrated in Figure 1, to distinguish: i) exposure-outcome confounders (variables that potentially confound the association between CSES and health and wellbeing in adulthood); ii) exposure-mediator confounders (variables that potentially confound the association between CSES and respondents’ education), and; iii) mediator-outcome confounders (variables that potentially confound the association between respondents’ education and health and wellbeing in adulthood). Age was considered a potential exposure-outcome confounder, mediator-outcome confounder, as well as an exposure-mediator confounder in all analyses. When childhood financial conditions was used as an exposure, fathers’ education, mothers’ education and spouse’s education were included in the model as potential mediator-outcome confounders. When mothers’ and fathers’ education were used as an exposure, childhood financial conditions and spouse’s education were included in the model as potential mediator-outcome confounders. Similarly, mothers’ education was included in the models when fathers’ education was used as an exposure, and fathers’ education was included in the model when mothers’ education was used as an exposure. Some of the models did not converge when age was used as a linear variable, therefore 5-year age groups were used in the analysis.

### Ethics approval

The Tromsø Study has been approved by the Regional Committee for Medical and Health Research Ethics, the Data Inspectorate and the Norwegian Directorate of Health.

## Results

The characteristics of the study sample are presented in Table 1. Half the sample (49.7%) were aged 60 years and above. Good or very good childhood financial conditions were reported among 72.4% of the respondents. There was a notable generational change in education. College or university education among parents was reported for only 5.9% of respondents’ mothers and 10.7% of respondents’ fathers, but for 37.7% of the respondents (Table 1).

The distribution of healthy respondents within each exposure and mediator category is presented in Table 2. The distribution of healthy respondents among those with low and high childhood financial conditions indicates that absolute differences were most apparent in self-rated health, subjective wellbeing, and the composite EQ-5D measure.

**Table 2:** **The proportion of healthy respondents in the study sample, and within each exposure and mediator category**

	% of healthy ^a^ respondents								
		Exposures (indicators of CSES)						Mediator	
Measures of subjective health and wellbeing	Total (n = 12,984)	Childhood financial conditions (n = 12,027)		Mothers’ education (n = 11,729)		Fathers’ education (n = 11,580)		Respondents’ education (n = 12,798)	
		Low n = 3317 (27.6%)	High n = 8710 (72.4%)	Low n = 9233 (78.7%)	High n = 2496 (21.3%)	Low n = 7435 (64.2%)	High n = 4145 (35.8%)	Non-university education n = 7962 (62.2%)	University education n = 4836 (37.8%)
Composite EQ-5D	44.6	34.6	48.4	42.2	54.1	41.6	50.4	38.1	54.8
EQ-5D health dimensions									
- Mobility	87.6	82.5	89.6	86.8	91.8	86.5	90.3	84.4	92.7
- Self-care	97.6	96.2	98.2	97.5	98.5	97.4	98.1	96.9	98.7
- Usual activities	85.1	78.6	87.5	84.2	89.5	83.9	87.7	81.8	90.4
- Pain/discomfort	49.7	40.6	53.2	47.1	60.5	46.5	56.2	42.9	60.9
Anxiety/depression	82.3	75.8	84.8	81.9	85.1	82.0	83.7	80.6	85.3
Self-rated health	65.8	55.2	70.7	64.3	76.8	63.0	74.1	59.2	77.2
Age-comparative self-rated health	30.3	28.6	31.4	29.2	36.2	28.9	34.0	25.6	37.9
Subjective wellbeing	36.4	25.9	40.3	35.3	40.3	35.5	38.0	33.8	39.9

^a^Health for EQ-5D was measured in three levels: level one ‘no problems’, level two ‘some problems’ level three ‘unable’ or having ‘extreme problems’. Healthy for composite EQ-5D and for all EQ-5D health dimensions included all respondents ticking level one for all five dimensions, or the single health dimension, respectively. Self-rated health was measured by the question “How do you in general consider your own health to be?”: very good, good, neither good nor bad, bad, and very bad. Healthy included those ticking very good or good. Age-comparative self-rated health was measured with the question “How do you consider your health compared to that of others your age?”: much better, somewhat better, about the same, a little worse, and much worse. Relatively healthy included those ticking the first two levels. Subjective wellbeing was measured by the first three items from the satisfaction with life scale measured on a 7-point scale. High wellbeing included those who reported 6 or 7 for all three items. CSES: childhood socioeconomic status.

Table 3 presents the NDEs, NIEs, and MTEs of childhood financial conditions on measures of subjective health and wellbeing separately for men and women. There was a null indirect association (NIE ≅ 1.00) of childhood financial conditions on measures of subjective health and wellbeing. The MTE is a product of the NDE and the NIE, so if the NIE ≅ 1.00, the NDE ≅ MTE. Consequently, the NDE and the MTE are similar in Table 3. Low childhood financial conditions led to a higher risk of being classified as unhealthy on all measures of subjective health and wellbeing, independent of respondents’ education. Among the five EQ-5D health dimensions, the absolute differences in four dimensions were small (Table 2), although the relative differences, expressed by RRs, were high (Table 3), e.g. self-care had a RR^MTE^ of 1.89 (95% CI: 1.11-3.23) for men, and 1.90 (95% CI: 1.22-2.96) for women. The dimension pain/discomfort showed the largest absolute difference in Table 2, but relatively low RRs in Table 3. RRs were not the same for men and women. Among men, childhood financial situation had a stronger effect on the composite EQ-5D measure (RR^MTE^ 1.22, 95% CI: 1.14-1.31), pain/discomfort dimensions (RR^MTE^ 1.21, 95% CI: 1.11-1.31), anxiety/depression dimension (RR^MTE^ 1.88, 95% CI: 1.57-2.25) and age-comparative self-rated health (RR^MTE^ 1.09, 95% CI: 1.04-1.15), but among women, childhood financial situation had a stronger effect on the self-care (RR^MTE^ 1.90, 95% CI: 1.22-2.96), usual activities (RR^MTE^ 1.67, 95% CI: 1.45-1.93), and as well as based on self-rated health (RR^MTE^ 1.45, 95% CI: 1.31-1.60).

**Table 3:** **The natural direct effects (NDE), natural indirect effects (NIE: mediated by respondents’ education) and marginal total effects (MTE) expressed as risk ratios (RRs) of childhood financial conditions on measures of subjective health and wellbeing**

Measures of subjective health and wellbeing	Childhood financial condition	NDE (RR) ^a^	95% CI	NIE (RR) ^a^	95% CI	MTE (RR) ^a^	95% CI
	Men (n = 3986)						
	High	1.00 (ref)	-	1.00 (ref)	-	1.00 (ref)	-
Composite EQ-5D	Low	1.22	1.14-1.31	1.00	1.00-1.01	1.22	1.14-1.31
EQ-5D health dimensions							
- Mobility	Low	1.20	0.97-1.49	1.01	0.98-1.04	1.21	0.97-1.51
- Self-care	Low	1.88	1.10-3.22	1.01	0.99-1.03	1.89	1.11-3.23
- Usual activities	Low	1.35	1.09-1.67	1.01	0.98-1.04	1.36	1.10-1.69
- Pain/discomfort	Low	1.20	1.11-1.31	1.00	1.00-1.01	1.21	1.11-1.31
- Anxiety/depression	Low	1.88	1.57-2.26	1.00	0.99-1.00	1.88	1.57-2.25
Self-rated health	Low	1.31	1.18-1.45	1.00	0.99-1.01	1.32	1.19-1.46
Age-comparative self-rated health	Low	1.09	1.03-1.14	1.00	0.99-1.01	1.09	1.04-1.15
Subjective wellbeing	Low	1.24	1.18-1.30	1.00	0.99-1.00	1.24	1.18-1.31
	**Women (n = 3974)**						
	High	1.00 (ref)	-	1.00 (ref)	-	1.00 (ref)	-
Composite EQ-5D	Low	1.16	1.10-1.23	1.00	0.98-1.01	1.16	1.10-1.22
EQ-5D health dimensions							
- Mobility	Low	1.83	1.54-2.18	0.99	0.98-1.01	1.82	1.53-2.17
- Self-care	Low	1.91	1.23-2.97	0.99	0.96-1.02	1.90	1.22-2.96
- Usual activities	Low	1.68	1.46-1.94	0.99	0.98-1.01	1.67	1.45-1.93
- Pain/discomfort	Low	1.13	1.07-1.21	1.00	0.98-1.01	1.13	1.07-1.20
- Anxiety/depression	Low	1.55	1.35-1.77	1.00	0.98-1.01	1.54	1.34-1.76
Self-rated health	Low	1.46	1.32-1.61	1.00	0.98-1.01	1.45	1.31-1.60
Age-comparative self-rated health	Low	1.03	0.99-1.07	1.00	0.99-1.01	1.03	0.99-1.07
Subjective wellbeing	Low	1.26	1.19-1.33	1.00	0.99-1.00	1.25	1.19-1.32

^a^Adjusted for age, spouse’s education, mothers’ education, and fathers’ education. NDE: Natural direct effects. NIE: Natural indirect effects. MTE: Marginal total effects. CI: confidence interval.

Table 4 presents the NDEs, NIEs, and MTEs of fathers’ education and mothers’ education on measures of subjective health and wellbeing. Among men, fathers’ education had almost a null effect (MTE/NDE/NIE ≅ 1.00) on subjective wellbeing. There was a protective effect of low fathers’ education on mobility (RR^MTE^ 0.77, 95% CI: 0.61-0.99). The decomposition into direct and indirect effects shows that there was an increased indirect risk, but a protective direct effect for mobility (RR^NIE^ 1.06, 95% CI: 1.02-1.11 vs RR^NDE^ 0.73, 95% CI: 0.57-0.93).

**Table 4:** **The natural direct effects (NDE), natural indirect effects (NIE: mediated by respondents’ education) and marginal total effects (MTE) expressed as risk ratios (RRs) of parental education on measures of subjective health and wellbeing**

Measures of subjective health and wellbeing		Parental education	NDE (RR)	95% CI	NIE (RR)	95% CI	MTE (RR)	95% CI
		Men (n = 3986)						
		High (ref)	1.00 (ref)	-	1.00 (ref)	-	1.00 (ref)	-
Composite EQ-5D	Mothers’ Education	Low	1.02^a^	0.92-1.13	1.02^a^	1.01-1.04	1.04^a^	0.94-1.15
	Fathers’ Education	Low	1.03^b^	0.94-1.12	1.02^b^	1.01-1.04	1.05^b^	0.96-1.15
EQ-5D health dimensions								
- Mobility	Mothers’ Education	Low	0.96^a^	0.72-1.30	1.09^a^	1.04-1.15	1.05^a^	0.78-1.42
	Fathers’ Education	Low	0.73^b^	0.57-0.93	1.06^b^	1.02-1.11	0.77^b^	0.61-0.99
- Self-care	Mothers’ Education	Low	0.93^a^	0.43-2.01	1.06^a^	0.95-1.18	0.99^a^	0.46-2.12
	Fathers’ Education	Low	0.59^b^	0.32-1.08	1.05^b^	0.95-1.15	0.61^b^	0.34-1.12
- Usual activities	Mothers’ Education	Low	1.15^a^	0.82-1.60	1.08^a^	1.03-1.14	1.24^a^	0.89-1.73
	Fathers’ Education	Low	0.98^b^	0.75-1.27	1.07^b^	1.03-1.12	1.05^b^	0.81-1.36
- Pain/discomfort	Mothers’ Education	Low	1.03^a^	0.92-1.16	1.03^a^	1.01-1.05	1.06^a^	0.95-1.20
	Fathers’ Education	Low	1.06^b^	0.96-1.17	1.03^b^	1.01-1.04	1.09^b^	0.99-1.20
- Anxiety/depression	Mothers’ Education	Low	0.93^a^	0.72-1.20	1.00^a^	0.96-1.03	0.93^a^	0.72-1.19
	Fathers’ Education	Low	0.99^b^	0.80-1.24	1.01^b^	0.98-1.04	1.00^b^	0.81-1.25
Self-rated health	Mothers’ Education	Low	1.11^a^	0.95-1.29	1.03^a^	1.01-1.06	1.14^a^	0.98-1.33
	Fathers’ Education	Low	0.98^b^	0.86-1.10	1.04^b^	1.02-1.06	1.01^b^	0.90-1.14
Age-comparative self-rated health	Mothers’ Education	Low	1.09^a^	1.01-1.16	1.03^a^	1.01-1.04	1.11^a^	1.04-1.19
	Fathers’ Education	Low	1.04^b^	0.98-1.10	1.02^b^	1.01-1.03	1.06^b^	1.01-1.12
Subjective wellbeing	Mothers’ Education	Low	1.04^a^	0.97-1.12	1.01^a^	1.01-1.02	1.05^a^	0.98-1.13
	Fathers’ Education	Low	1.00^b^	0.94-1.06	1.01^b^	1.00-1.02	1.01^b^	0.95-1.07
		**Women (n = 3974)**						
		High (ref)	1.00 (ref)	-	1.00 (ref)	-	1.00 (ref)	-
Composite EQ-5D	Mothers’ Education	Low	1.10^a^	1.02-1.19	1.03^a^	1.02-1.05	1.14^a^	1.05-1.23
	Fathers’ Education	Low	0.98^b^	0.92-1.04	1.03^b^	1.02-1.05	1.01^b^	0.95-1.07
EQ-5D health dimensions								
- Mobility	Mothers’ Education	Low	1.00^a^	0.77-1.30	1.05^a^	1.01-1.10	1.05^a^	0.81-1.37
	Fathers’ Education	Low	0.83^b^	0.67-1.02	1.05^b^	0.99-1.10	0.86^b^	0.70-1.06
- Self-care	Mothers’ Education	Low	0.65^a^	0.35-1.22	1.06^a^	0.95-1.18	0.69^a^	0.37-1.28
	Fathers’ Education	Low	0.83^b^	0.48-1.44	1.07^b^	0.94-1.21	0.88^b^	0.52-1.52
- Usual activities	Mothers’ Education	Low	1.02^a^	0.82-1.25	1.05^a^	1.01-1.09	1.07^a^	0.86-1.32
	Fathers’ Education	Low	0.88^b^	0.74-1.05	1.07^b^	1.02-1.11	0.94^b^	0.80-1.11
- Pain/discomfort	Mothers’ Education	Low	1.12^a^	1.03-1.22	1.04^a^	1.02-1.06	1.16^a^	1.07-1.27
	Fathers’ Education	Low	0.97^b^	0.91-1.04	1.04^b^	1.02-1.05	1.01^b^	0.94-1.08
- Anxiety/depression	Mothers’ Education	Low	1.38^a^	1.13-1.69	1.05^a^	1.02-1.08	1.45^a^	1.18-1.78
	Fathers’ Education	Low	0.85^b^	0.72-0.98	1.05^b^	1.02-1.09	0.89^b^	0.76-1.03
Self-rated health	Mothers’ Education	Low	1.02^a^	0.87-1.19	1.08^a^	1.05-1.12	1.10^a^	0.94-1.29
	Fathers’ Education	Low	1.09^b^	0.96-1.23	1.08^b^	1.04-1.11	1.17^b^	1.03-1.32
Age-comparative self-rated health	Mothers’ Education	Low	1.04^a^	0.99-1.09	1.02^a^	1.01-1.03	1.06^a^	1.01-1.11
	Fathers’ Education	Low	1.02^b^	0.98-1.06	1.02^b^	1.01-1.03	1.04^b^	1.00-1.08
Subjective wellbeing	Mothers’ Education	Low	1.04^a^	0.96-1.12	1.01^a^	0.99-1.02	1.04^a^	0.96-1.13
	Fathers’ Education	Low	0.95^b^	0.89-1.01	1.00^b^	0.99-1.02	0.95^b^	0.90-1.01

^a^Adjusted for age, spouse’s education, childhood financial conditions, and fathers’ education. ^b^Adjusted for age, spouse’s education, childhood financial conditions, and mothers’ education.

NDE: Natural direct effects. NIE: Natural indirect effects. MTE: Marginal total effects. CI: confidence interval.

Among men, low mothers’ education increased the risk of being unhealthy on age-comparative self-rated health (RR^MTE^ 1.11, 95% CI: 1.04-1.19). There was an increased indirect (NIEs) risk for composite EQ-5D, mobility, usual activities, pain/discomfort, self-rated health, age-comparative self-rated health, and subjective wellbeing. However, for anxiety/depression there was no indirect effect (RR^NIE^ 1.00, 95% CI: 0.96-1.03), and consequently the NDE was almost the same as the MTE (RR 0.93, 95% CI: 0.72-1.20).

Among women, low fathers’ education increased the risk of being unhealthy on self-rated health (RR^MTE^ 1.17, 95% CI: 1.03-1.32). The decomposition of MTEs into direct and indirect effects shows that there was an increased indirect risk (NIEs) for composite EQ-5D, usual activities, pain/discomfort, anxiety/depression, self-rated health and age-comparative self-rated health. However, there was a protective direct effect for anxiety/depression (RR^NDE^ 0.85, 95% CI: 0.72-0.98). Low mothers’ education increased the risk of being unhealthy on composite EQ-5D (RR^MTE^ 1.14, 95% CI: 1.05-1.23, pain/discomfort (RR^MTE^ 1.16, 95% CI: 1.07-1.27), anxiety/depression (RR^MTE^ 1.45, 95% CI: 1.18-1.78), and age-comparative self-rated health (RR^MTE^ 1.06, 95% CI: 1.01-1.11). The decomposition of MTEs into direct and indirect effects shows that there was an increased indirect risk (NIEs) for composite EQ-5D, mobility, usual activities, pain/discomfort, anxiety/depression, self-rated health, and age-comparative self-rated health. However, there was an increased direct risk (NDEs) for composite EQ-5D, pain/discomfort, and anxiety/depression.

Table 5 presents the CDEs of childhood financial conditions, fathers’ education, and mothers’ education on health and wellbeing measures controlled separately at both levels of respondent’s education. Among both men and women, having low childhood financial conditions increased the risk of being unhealthy on almost all health and wellbeing measures, regardless of the level of respondents’ education.

**Table 5:** **The controlled direct effects (CDE) expressed as risk ratios (RRs) of childhood financial conditions, mothers’ education, and fathers’ education on measures of subjective health and wellbeing by respondents’ education**

Measures of subjective health and wellbeing	CSES		Low respondents’ education		High respondents’ education	
			CDE (RR)	95% CI	CDE (RR)	95% CI
		Men (n = 3986)				
		High	1.00 (ref)	-	1.00 (ref)	-
Composite EQ-5D	Childhood financial condition	Low	1.18^a^	1.09-1.29	1.28^a^	1.13-1.44
	Mothers’ Education	Low	0.94^b^	0.82-1.07	1.09^b^	0.95-1.25
	Fathers’ Education	Low	1.03^c^	0.92-1.14	1.03^c^	0.91-1.17
EQ-5D health dimensions						
- Mobility	Childhood financial condition	Low	1.24^a^	0.97-1.59	1.12^a^	0.73-1.71
	Mothers’ Education	Low	0.91^b^	0.62-1.33	1.04^b^	0.69-1.58
	Fathers’ Education	Low	0.67^c^	0.51-0.89	0.82^c^	0.55-1.22
- Self-care	Childhood financial condition	Low	1.63^a^	0.88-3.01	2.62^a^	0.95-7.19
	Mothers’ Education	Low	0.55^b^	0.24-1.28	3.45^b^	0.76-15.74
	Fathers’ Education	Low	0.45^c^	0.23-0.88	0.95^c^	0.33-2.74
- Usual activities	Childhood financial condition	Low	1.56^a^	1.23-1.98	0.99^a^	0.65-1.53
	Mothers’ Education	Low	1.16^b^	0.75-1.80	1.13^b^	0.72-1.76
	Fathers’ Education	Low	1.04^c^	0.77-1.42	0.90^c^	0.60-1.35
- Pain/discomfort	Childhood financial condition	Low	1.15^a^	1.05-1.27	1.28^a^	1.11-1.47
	Mothers’ Education	Low	0.94^b^	0.81-1.10	1.13^b^	0.96-1.32
	Fathers’ Education	Low	1.04^c^	0.93-1.17	1.08^c^	0.94-1.25
- Anxiety/depression	Childhood financial condition	Low	1.77^a^	1.40-2.24	2.02^a^	1.54-2.64
	Mothers’ Education	Low	0.99^b^	0.69-1.45	0.89^b^	0.66-1.20
	Fathers’ Education	Low	1.20^c^	0.90-1.61	0.85^c^	0.64-1.14
Self-rated health	Childhood financial condition	Low	1.30^a^	1.16-1.46	1.33^a^	1.11-1.60
	Mothers’ Education	Low	0.92^b^	0.76-1.11	1.36^b^	1.10-1.68
	Fathers’ Education	Low	0.98^c^	0.85-1.13	0.97^c^	0.81-1.17
Age-comparative self-rated health ^b^	Childhood financial condition	Low	1.06^a^	1.01-1.12	1.13^a^	1.03-1.23
	Mothers’ Education	Low	1.04^b^	0.96-1.13	1.13^b^	1.02-1.24
	Fathers’ Education	Low	1.04^c^	0.98-1.11	1.03^c^	0.95-1.13
Subjective wellbeing	Childhood financial condition	Low	1.20^a^	1.13-1.28	1.29^a^	1.19-1.40
	Mothers’ Education	Low	0.95^b^	0.86-1.04	1.13^b^	1.02-1.24
	Fathers’ Education	Low	0.96^c^	0.90-1.03	1.04^c^	0.95-1.14
		**Women (n = 3974)**				
		High	1.00 (ref)	-	1.00 (ref)	-
Composite EQ-5D	Childhood financial condition	Low	1.19^a^	0.13-1.25	1.11^a^	0.99-1.24
	Mothers’ Education	Low	1.02^b^	0.92-1.13	1.20^b^	1.07-1.34
	Fathers’ Education	Low	0.93^c^	0.87-0.99	1.04^c^	0.94-1.16
EQ-5D health dimensions						
- Mobility	Childhood financial condition	Low	1.91^a^	1.58-2.30	1.68^a^	1.16-2.43
	Mothers’ Education	Low	0.85^b^	0.61-1.18	1.27^b^	0.87-1.86
	Fathers’ Education	Low	0.73^c^	0.58-0.93	0.97^c^	0.68-1.39
- Self-care	Childhood financial condition	Low	2.24^a^	1.40-3.57	1.31^a^	0.48-3.56
	Mothers’ Education	Low	0.61^b^	0.28-1.35	0.71^b^	0.29-1.70
	Fathers’ Education	Low	0.84^c^	0.45-1.54	0.82^c^	0.33-2.01
- Usual activities	Childhood financial condition	Low	1.79^a^	1.54-2.09	1.46^a^	1.08-1.96
	Mothers’ Education	Low	0.95^b^	0.72-1.25	1.11^b^	0.84-1.48
	Fathers’ Education	Low	0.91^c^	0.75-1.11	0.85^c^	0.65-1.13
- Pain/discomfort	Childhood financial condition	Low	1.16^a^	1.09-1.24	1.08^a^	0.94-1.23
	Mothers’ Education	Low	1.04^b^	0.93-1.17	1.21^b^	1.07-1.37
	Fathers’ Education	Low	0.91^c^	0.84-0.98	1.06^c^	0.94-1.19
- Anxiety/depression	Childhood financial condition	Low	1.63^a^	1.40-1.91	1.38^a^	1.07-1.78
	Mothers’ Education	Low	1.37^b^	1.02-1.85	1.39^b^	1.09-1.78
	Fathers’ Education	Low	0.84^c^	0.70-1.01	0.84^c^	0.66-1.06
Self-rated health	Childhood financial condition	Low	1.44^a^	1.31-1.60	1.48^a^	1.19-1.84
	Mothers’ Education	Low	1.07^b^	0.87-1.32	0.96^b^	0.77-1.19
	Fathers’ Education	Low	1.07^c^	0.93-1.22	1.12^c^	0.91-1.38
Age-comparative self-rated health	Childhood financial condition	Low	1.04^a^	1.00-1.09	1.01^a^	0.93-1.10
	Mothers’ Education	Low	0.99^b^	0.95-1.05	1.08^b^	1.01-1.17
	Fathers’ Education	Low	0.99^c^	0.95-1.03	1.05^c^	0.98-1.13
Subjective wellbeing	Childhood financial condition	Low	1.28^a^	1.20-1.36	1.23^a^	1.11-1.35
	Mothers’ Education	Low	1.03^b^	0.92-1.15	1.04^b^	0.95-1.14
	Fathers’ Education	Low	0.93^c^	0.86-1.00	0.97^c^	0.89-1.07

^a^Adjusted for age, spouse’s education, mothers’ education, and fathers’ education. ^b^Adjusted for age, spouse’s education, childhood financial conditions, and fathers’ education. ^c^Adjusted for age, spouse’s education, childhood financial conditions, and mothers’ education.

CDE: Controlled direct effects. CI: confidence interval.

Among men, there was an increased CDE^High respondents’ education^ of low mothers’ education on self-rated health, age-comparative self-rated health, and subjective wellbeing (Table 5). However, there was a protective direct effect (CDE^Low respondents’ education^) of low fathers’ education on mobility (RR^CDE^ 0.67, 95% CI: 0.51-0.89) and self-care (RR^CDE^ 0.45, 95% CI: 0.23-0.88).

Among women, there was an increased CDE of low mothers’ education on anxiety/depression, regardless of the level of the respondents’ education controlled. There was an increased CDE^High respondents’ education^ of low mothers’ education on composite EQ-5D (RR^CDE^ 1.20, 95% CI: 1.07-1.34), pain/discomfort (RR^CDE^ 1.21, 95% CI: 1.07-1.37), and age-comparative self-rated health (RR^CDE^ 1.08, 95% CI: 1.01-1.17). However, there was a protective direct effect (CDE^Low respondents’ education^) of low fathers’ education on composite EQ-5D (RR^CDE^ 0.93, 95% CI: 0.87-0.99), mobility (RR^CDE^ 0.73, 95% CI: 0.58-0.93), and pain/discomfort (RR^CDE^ 0.91, 95% CI: 0.84-0.98) (Table 5).

## Discussion

We estimated the effects of childhood financial conditions, fathers’ education, and mothers’ education on several measures of health and wellbeing. These total effects are further decomposed into direct and indirect effects, which allowed us to analyze the mediating role of respondents’ education. As all the three exposures were adjusted for one another, our results aim to present the unique effect of each indicator of CSES, and not the cumulative effect of CSES on health and wellbeing in adulthood.

Our results show that childhood financial conditions have a strong direct effect on health and wellbeing in adulthood, independent of respondents’ education, while generally speaking parental education has an indirect effect on health and wellbeing in adulthood, mediated by respondents’ education. This indicates that effect of childhood financial conditions on health and wellbeing in adulthood is long-term, and that there may be other pathways from childhood financial conditions to health and wellbeing besides respondents’ education. However, the effect of parental education on later health and wellbeing was not independent of respondents’ education.

Childhood financial conditions reflect only economic conditions, and educated parents are not necessarily wealthy during the early childhood of their offspring. A substantial proportion of parents may have completed their education after their child had grown up. The difference between the effects of childhood financial conditions and parental education may highlight this difference. For children, the strongest contribution of parental education may be the inspiration, motivation, and guidance in achieving higher education. However, the potential mechanisms of childhood financial conditions that lead to health and wellbeing in adulthood may be the better living conditions, and availability of resources from an early age.

Our study confirms that the effect of parental education on health in adulthood is mediated by ASES. However, there are some indications that mothers’ education has both a direct (i.e. independent of respondents’ education) and an indirect effect on health in adulthood. Low mothers’ education led to an increased risk (NDE) in women for being unhealthy on the composite EQ-5D, pain/discomfort, and the anxiety/depression dimension. While among men, having low mothers’ education increased the direct risk (NDE) of being unhealthy on age-comparative self-rated health.

Some limitations should be considered when interpreting the results of this study. The estimation of NDEs, NIEs , and the causal interpretation require that there be no unmeasured exposure-mediator confounders, and that no mediator-outcome confounder be effected by the exposure [42]. Both of these assumptions seem unrealistic given the limited set of covariates we included in the models. For instance, the CSES is likely to affect the health of the respondent in childhood, which in turn is likely to affect both ASES and health in adulthood. Similarly, parental health is likely to affect both CSES, and respondents’ education. Therefore, CDEs are also reported. However, for a causal interpretation of the CDEs, there must be no unmeasured exposure-outcome confounder, and no unmeasured mediator-outcome confounder [42]. Some of the potential mediator-outcome confounders that are missing in the analysis are ‘health of the respondent in childhood’ and neighborhood. Similarly, parental mental and physical health are potential exposure-outcome confounders missing in the analysis. In the absence of these confounders, the causal interpretation of estimates is not realistic.

Many of the previous studies [4, 5, 7, 9, 11, 12, 19] have assessed the mediating role of ASES in the association between CSES and later health and wellbeing by difference method approach. In this method, the outcome is regressed on the exposure, conditional on the covariates, and then the assumed mediator is added to the model to assess whether there was a reduction in the estimate for the exposure. However, the assumptions needed for the causal interpretation of the estimates from the difference method approach are same, as the counterfactual approach we have used. Therefore, the same criticism of whether these estimates can be interpreted as causal, applies also to most previous studies using data from observational studies. The ‘no unmeasured confounding’ assumptions can only be satisfied successfully if both the exposure and mediator were randomized. Moreover, there are two more limitations in using the difference method approach. Firstly, if there was an exposure-mediator interaction, the difference method provides biased estimates[43]. Secondly, if the outcome is not rare, the odds ratio is not a suitable measure for assessing mediation with the difference method approach [39, 44]. Several previous studies [7, 9, 11, 12, 19] have used the difference method approach in logistic regression (ORs) when the outcome was not rare. Similarly, the application of linear structural equation modelling framework is not generalizable to nonlinear models to assess mediation [44–46]. The strength of this paper is that we provide NDEs, NIEs and CDEs, in the presence of exposure-mediator interaction; thus highlighting the effect of interaction, and the RR estimates are given when the outcome was not rare.

For each indicator of CSES, two CDEs are reported. Both are interpreted as direct effects of CSES unmediated by respondents’ education. The selection of a precise value of a mediator is crucial in circumstances where the CDEs vary greatly. This would depend on the magnitude of the exposure-mediator interaction term and the plausibility of imagining a world where everyone had a fixed level of the mediator. Both the CDEs we report for the effect of childhood financial conditions are in the same direction. They are also similar to the corresponding NDEs, as the NDEs can be seen as the weighted average of the CDEs [44]. However, the direction of the CDEs varies when the effect of parental education on health and wellbeing was analyzed. This shows that the effect of parental education on health and wellbeing depends strongly on the level of respondent’s education.

Our measurement of CSES indicators was based on recall. Recall bias may have led to an overestimation or underestimation of the associations. A previous study showed that recall of fathers’ education is accurate [34]; however we could not find any study where the reliability and validity of childhood financial conditions was reported. Among the indicators of CSES, the variable childhood financial conditions had the fewest missing values, which is consistent with a previous study assessing the pattern of missing data across various indicators of CSES [47]. This may indicate that, apart from the possibility of recall bias, the respondents may not know the highest education level of their parents. Recall of CSES indicators may be effected by “an inability to remember, refusal to answer, embarrassment in answering or lack of information about early-life circumstances” [47]. There is ample evidence that state of mind effects certain aspects of memory [48], and therefore the possibility of recall bias cannot be ruled out.

The classification we used for education may not apply accurately to respondents of different age groups. For example, respondents with an education of college/university less than 4 years may have been considered highly educated in the 1960s, but not in the 1990s. We acknowledge that our assumption of temporality between the CSES, respondents’ education, and subjective health and wellbeing in adulthood is based on a conceptual model. Since the data is cross-sectional in nature, this may present a possible bias in our study. For example, among the youngest respondents (aged 30–35 years), the assumed temporality between their education and their health may not be precise, as some may still be studying part-time.

Respondents with missing values on any of the variables in the statistical models were excluded from the analysis. We assessed whether no response (missing) on the CSES indicators was related to health and wellbeing indicators, and the analysis showed that a greater proportion of those who did not provide a response on CSES indicators had low education, and were relatively unhealthy (particularly in relation to parental education) (data not shown). We also assessed whether no response (missing) on the health and wellbeing indicators was related to CSES indicators, but the pattern was same. A greater proportion of those who do not respond to health and wellbeing questions have low CSES (data not shown). This may indicate that those who do not complete the questionnaire are likely to be the most disadvantaged. However, if we had the data on all respondents, it is likely that the estimates (NDEs/CDEs) would show an even larger effect of childhood financial conditions on health and wellbeing, in the same direction as shown. Similarly, it seems plausible that we would observe a clear association between low parental education and being unhealthy/low wellbeing if we had the data on all respondents. Since the missing data is not random, it is likely that imputation will introduce more uncertainty, and bias in our results. Therefore, we chose to analyze the collected data only.

We estimated the CIs for NDEs, NIEs and MTEs in all analysis with bootstrapping, but there were no meaningful differences in the CIs even with large number of replications. Therefore we did not use bootstrapping in the analysis. Some studies [49, 50] have reported the ‘proportion mediated’ [39, 46], ‘% excess risk explained’ [51], or a conceptually similar measure to distinguish the proportion or percentage of the indirect effect from total effects. We did not report the ‘proportion mediated’, as many of the NIEs were not statistically significant, and because the direction of NDEs and NIEs was not the same for many measures of health and wellbeing when mothers’ education and fathers’ education were used as an exposure.

Previous research on the interaction between CSES and ASES, and its effect on health in adulthood is not consistent [7, 16, 21, 49, 52]. We have presented CDEs to highlight the influence of exposure-mediator interaction, and the role of respondents’ education as a moderator in our data. The potential weakness is that respondents’ education is merged into two groups, and there may be heterogeneity within each group. We did not assess the mediating role of other adult SES indicators. The methodological challenge in assessing the mediating role of income or occupation is that respondents’ education is likely to be a mediator-outcome confounder affected by the CSES.

It is generally assumed that self-rated health is insensitive to the wording used in the question [53]. Our results suggest that self-rated health and age-comparative self-rated health do not measure subjective health in a similar manner. This is probably because the comparison group was not determined in the question on non-comparative self-rated health. The respondents may have compared their health with others of same sex, or their health at other times, and their response could have been influenced by the expectations others have of their health [54]. Some research [55] suggests that the agreement between the non-comparative self-rated health and age-comparative self-rated health may be excellent in some age groups. However, we observed that the NDEs and NIEs of CSES for both self-rated health measures were not similar across age groups (data not shown). One plausible explanation for the difference may be that for age-comparative self-rated health, the respondents compared their health to peers who likely have similar socio-economic status. As the health profile is more similar among people from the same socio-economic groups, the respondents may not compare with the health status in the wider population outside of their own reference group.

Although the previous research exploring the causal mechanisms of the effect of parental education and income on adult health is not consistent, Deaton [40] summarized some of the previous research and proposed that the effect of parental education on adult health is likely to be mediated by both parents’ income, and respondents’ education. Our data from Norway suggests that the effect of parental education on adult health is mediated by respondents’ education. Similarly, Deaton [40] proposed that the effect of parents’ income on adult health is likely to mediate through respondents’ education, but our data shows that the effect of childhood financial conditions is not mediated by respondents’ education. This may be due to the egalitarian nature of Norwegian society.

Previous studies have shown that among different measures of ASES, education is a main mediator between CSES and later health [17, 56]. Our findings suggest that the mediating role of respondents’ education is different according to the indicators of CSES used in the analysis. In contrast to most previous studies, where both a direct and indirect effect (mediated by ASES) of CSES were observed [4, 6, 12], our study showed no evidence of a mediating effect for respondents’ education when childhood financial conditions was used as an exposure. However, we observed little evidence of either a direct or an indirect effect of parental education on some health measures. Many studies [2, 8–11, 21] have indicated that most of the effect of parental education on health and wellbeing in adulthood is mediated by adult SES. Our findings support this.

One interesting finding from previous studies is that mothers’ education is more important than the fathers’ education on adult health [2]. This probably reflects the less dominant role of fathers in child rearing, and looking after children’s health [2, 57]. It is uncertain whether this trend will continue. Longitudinal studies assessing the effect of parental education on later health in different generations are needed to explore this further.

Several studies have assessed the effect of CSES on indicators of psychological symptoms, but yielded inconsistent results [10, 16, 21, 22, 24, 26, 27, 58]. Most studies have found evidence of a direct effect [1, 7, 16, 21, 22, 24], while others have found evidence of an indirect effect [10, 26]. We have found evidence of a direct effect of childhood financial conditions and mothers’ education on anxiety/depression, as well as an indirect effect of mothers’ education on anxiety/depression among women.

## Conclusions

Our findings suggest that childhood financial conditions have a unique direct effect on a wide range of health and wellbeing measures. These findings apply to both men and women. Generally, parental education has an indirect effect on later health, but mothers’ education may also have a long-term direct effect on later health. Consistent with previous research on the effects of CSES on cause specific mortality and morbidity, our results suggest that, in addition to effecting adult SES, many aspects of subjective health and wellbeing may also have socio-economic roots in childhood.
